# An overview of viral oncology in Italy - report from the Pavia meeting on solid tumors

**DOI:** 10.1186/1750-9378-7-23

**Published:** 2012-09-05

**Authors:** Vittorio Perfetti, Mattia Ricotti, Franco Buonaguro, Umberto Tirelli, Paolo Pedrazzoli

**Affiliations:** 1 Fondazione IRCCS Policlinico “San Matteo”, Pavia, Italy; 2 National Cancer Institute, IRCCS Fondazione “G. Pascale”, Napoli, Italy; 3 National Cancer Institute, Aviano, Italy

## Abstract

This is a report on some of the research activities currently ongoing in Italy as outlined at the “Viruses and solid tumors” meeting jointly organized by the Oncology Sections of IRCCS Policlinico “San Matteo” (Pavia) and IRCCS National Cancer Institute (Aviano), held in Pavia, Italy, on October 2011. Experts from the various disciplines involved in the study of the complex relationships between solid tumors and viruses met to discuss recent developments in the field and to report their personal contributions to the specified topics. Secondary end point was to establish a multidisciplinary work group specifically devoted to solid tumors and infectious agents, aimed to identify areas of common interest, promoting and establishing collaborative projects and programs, and to coordinate clinical and research activities. The group, which will be named IVOG (Italian Viral Oncology Group), will operate under the patronage of the various scientific societies of interest.

## Of viruses and tumors, general aspects

The first introductory lecture dealt with the fundamental milestones of the long lasting and successful research on viruses and cancer, such as the discovery of transforming non-filterable agents that cause cancer, the identification of the first human tumor virus (EBV), the discoveries of proto-oncogenes (*SRC*) and tumor suppressor genes (*p53*, *Rb*), and the first vaccination achievements (HBV, HPV) (Umberto Veronesi, Istituto Europeo di Oncologia, Milan, Italy). Then it was the turn to address the intimate mechanisms of direct and indirect virus oncogenesis, illustrating the key steps and complex regulatory circuits that contribute to the cancer development (Giorgio Palù, University of Padova, Italy). At the end, Antonino Carbone (National Cancer Institute, Aviano, Italy) reviewed the molecular techniques for pathologic and virologic investigations, including DNA/RNA detection techniques and conventional immunohistochemistry for EBV, KSHV, MCPyV and HPV.

## Epidemiology

Epidemiology of virus-associated cancers was introduced by Diego Serraino (IRCCS Centro di Riferimento Oncologico, Aviano, Italy) who presented an interesting study on HLA-A and -B high-resolution profile in EBV-associated undifferentiated nasopharyngeal carcinoma (UNPC) in Italy, a non endemic area for this disorder [1]; it was shown that affected individuals present distinct HLA- genetic profile, with significant hyper- and under-representation of specific alleles, compared to bone marrow donors. As for other virus-driven tumors, the lack of an HLA background favoring an efficient immune control of EBV-infected cells may increase the risk to develop nasopharyngeal carcinoma, with data that are relevant for immunotherapy (EBV specific CTLs and vaccines) in these areas.

The estimated total number of cancer cases attributable to infection in the year 2002 was 1.9 million, or 17.8% of the global cancer burden [2]. The attributed fraction at the specific sites varied from 100% of cervical cancers attributed to the papilloma viruses to a tiny proportion (0.4%) of liver cancers (worldwide) caused by liver flukes. Analysis of a number of cancer cases attributed to infection by age group indicated that in developing countries virus-associated cancers cluster in the 50–69 age group. Silvia Franceschi, the epidemiologist director of the Infection and Cancer Epidemiology Group at the WHO/OMS International Agency for Research on Cancer (IARC, Lyon, France), focused the attention on HPV as a good example of a carcinogenic infection with the typical features of an etiological association between infections and human cancers. Data from the IARC Prevalence Surveys, 1990–2011, indicated that the prevalence of cervical HPV DNA in Italy was about 9%. Franceschi presented a recent meta-analysis of HPV type-specific prevalence data published from 1990 to 2010, including a total of 243 studies and 30,848 invasive cervical cancers (ICC) [3]. Results showed that the proportion of ICC associated with HPV-16 and HPV-18 was between 70 and 76% in all world regions except for Asia. The 12 most common HPV types identified, in order of decreasing prevalence, were HPV-16 (57%), 18 (16%), 58, 33, 45, 31, 52, 35, 59, 39, 51 and 56. The prevalence of other types, phylogenetically related to those above, ranged from <0.1% for HPV 85 to 0.6% for HPV-68. There was a significant increase in the prevalence of multiple infections (from 4.0%, year 1990, to 15.7%, year 2010) and of HPV-16 (from 51.8 to 60.0%). Smaller, but relevant increases were also seen for HPV-39, 53 and 58. According to recent data, the fraction of anus cancer attributable to HPV infection raised to 88%.

In recent studies the involvement of HPV infection in head and neck cancers was investigated. Data indicated that the fraction of oropharynx, tonsils and base of tongue cancers attributed to HPV was 50% in developed countries and 5% in developing countries (where smoke and alcohol were the major risk factors). The natural history of cervical HPV infection is well studied, whereas the extension of cervical findings to HPV carcinogenesis in the tonsil needs more investigations. Cervical HPV infection is mainly transmitted through sexual intercourse, and is very common and rising in all world population. On the other hand, information on the prevalence and the age at first acquisition of HPV infection to the oropharynx is limited in quantity and quality. HPV-16 was found in 50% of cervical carcinoma and in 90% of HPV-positive carcinomas of the oropharynx. The precancerous HPV-associated lesions that precede cervical carcinoma (CIN1-3) are well defined and widely employed in screening programs. In contrast, precancerous lesions in the oropharynx are poorly understood and not consistently classified. Contrary to cervical carcinoma, very strong risk factors, other than HPV, exist for oropharyngeal carcinoma (mainly smoke and alcohol). In conclusion, Franceschi presented the future objectives emerged from the FP-7 funded IARC Study of human Papillomavirus and precancerous Lesions In the Tonsil (SPLIT study): i) prevalence of HPV infection in cancer-free tonsils and age at acquisition; ii) detection of precancerous lesions in the tonsil using histological and cytological examinations; iii) differences between HPV-related precancerous lesions in the oropharynx and those caused by other risk factors (smoke, alcohol); iiii) fraction of oropharyngeal carcinomas truly attributable to HPV, in different countries.

## Cancers related to HPV and hepatitis viruses (HBV and HCV)

### Head and neck (HN) cancers

HPV 16, and secondarily HPV 18, are by far the most relevant human Papillomavirus types involved in oropharyngeal, laryngeal and oral cavity cancer, being observed in a relevant fraction of HN cancers, particularly in oropharyngeal tract. A role cannot be excluded for HPV types 6 and 11 too, since a minority of cancers (less than 5-10%) can be demonstrated to harbor sequences from these types. Several studies reported that detection of HPV DNA is associated with poorly differentiated cancer grade, positive lymph nodes, and late-stage disease, which traditionally indicate poor prognosis. Despite these observations, the outcome of HPV-positive tumors, progression-free survival (PFS) and overall survival (OS), appears to be significantly better than HPV negative tumors [4]. The more favorable prognosis of HPV-tumors was confirmed by a study conducted at the National Cancer Institute of Milan [5], based on a retrospective series of 90 consecutive oropharyngeal cancer patients treated primarily with surgery. Results showed that HPV-positive status (19/90, all positive for HPV-16 genotype) was associated with improved overall survival, reduced incidence of tumor relapse and second tumors. The reasons for the more favorable prognosis of HPV-positive tumors remain unknown; there are some biological elements typical of these tumors, such as lower mutational rate and less complex chromosomal abnormalities, which may help to understand this issue. The less complex genomic alterations may indicate that HPV tumors are at an earlier step in the cancerogenesis evolution, “younger” in comparison with their HPV-negative counterparts, and therefore more easily treated and characterized by lower recurrence rates. Marco Merlano (A.O. Santa Croce e Carle, Cuneo, Italy) stated that there is now sufficient evidence to consider determination of HPV status mandatory in HN cancers, given its major relevance as prognostic and predictive marker.

Overexpression of p16, a cell cycle regulator, in HPV-positive oropharyngeal squamous cell carcinoma results from degradation of pRb by the viral oncoprotein E7; for this reason, p16 positivity at immunohistochemistry has been proposed as a surrogate marker for high-risk HPV infection. However, although some studies indicated a good correspondence between p16 and HPV status (assessed by DNA-ISH or mRNA E6/E7 Real- time PCR), a recent study from the Licitra group in Milan [6], evaluating an algorithm for the definition of HPV status in oropharyngeal cancer, demonstrated the existence of a cancer subset characterized by p16-positivity and consistently negative HPV-ISH. These data clearly indicate that evaluation of HPV via molecular biology is presently necessary.

### Cervical cancer

The WHO/ICO 2010 summary report on HPV and Cervical Cancer (HPV Information Centre) indicated 2880 new cervical cancer cases/year in Italy, with a crude incidence rate of about 9.4 per 100,000 women/year, and with a prevalence of HPV-16 and/or HPV-18 among women with high grade cervical lesions (HSIL, CIN2, CIN3 and CIS) of 55.7%, and with cervical cancer of 72.1%. The crude incidence rate observed in Italy (9.4 per 100,000 women/year) indicated a slightly lower incidence in comparison to other Southern Europe countries (11.1%), but significantly lower compared to the global overall incidence (15.8%). Arsenio Spinillo (University of Pavia-Fondazione IRCCS Policlinico “San Matteo”, Pavia, Italy) presented studies on multiple HPV strain infection in cervical specimens. HPV DNA was investigated by means of a robust and sensitive PCR method for the simultaneous detection of multiple HPV types (SFP_10_-LIPA, detecting 15 high-risk and 10 low-risk HPV types) on a consecutive series of 1323 women undergoing colposcopy, 69% of whom had a cervical biopsy, and correlated CIN severity with the type and number of HPV [7]. The prevalence of HPV DNA was 97.3% in CIN 1 and 98.1% in CIN ≥ 2. Infection by multiple HPV types was observed in the majority of cases (75%). The frequency of multiple viral type detection increased in high-grade lesions (63.1% CIN1 and 80.8% CIN ≥ 2), with HPV-16 present, together with other types, in approximately half of the cases. Twenty-three different types were detected, HPV-16, 31, 51 being the most frequent. Number of viral types and class of oncogenic risk were linearly correlated with CIN severity by univariate and multivariate analyses. In this Italian series, multiple infection *per se* was associated with high-grade lesions, independently of HPV-16, stressing the need to study the role of this phenomenon in cervical precancerous lesions.

To study the distribution of HPV types in Northern Italy over the years 1985–2007, the Pavia group validated the SPF_10_ LiPA HPV typing assay in archival formalin fixed paraffin embedded tissue [8]. Results showed important changes over the last decade, with a reduction of the frequency of HPV 16/ 18 (HPV-16 decreased from 72 to 59%). Features of present infections were multiple genotype infections (up to 60% in high-grade lesions) and new HPV types (HPV-51, 52, 53, 56, 58, 66). The prevalence of these emerging genotypes was distributed over a wide range (5.7-30.8%) [9]. HPV-16 was the commonest genotype both in single and multiple infections.

Giorgio Palù presented preliminary data from his group on miRNAs expression in HPV associated cervical cancer. miRNAs are a new class of molecules recently studied for their possible role in oncogenesis and cancer progression. Indeed, miRNAs expression is significantly altered in many cancers, including cervical cancer. By miRNAs microarray and real-time PCR assays, Palù and collaborators identified cellular miRNAs significantly associated with HPV-16 E6 and E7 expression in human foreskin fibroblasts and keratinocytes. The relevant miRNAs appeared to be involved in the induction of cell proliferation, cell differentiation, invasiveness, apoptosis suppression and inhibition of IFN response; some of these miRNAs were up-regulated, while others were down-regulated. Successively, miRNAs expression was investigated in cervical specimens of 120 subjects enrolled for cervical cancer screening. Results demonstrated that these same miRNAs were up-regulated or down-regulated also *in vivo* and not only *in vitro*, attesting the relevance of this ongoing research.

### HCC

HCC is the 5^th^ most common cancer in men and 7^th^ one in women, and is the most frequent cause of death in patients with HCV-related cirrhosis in developing countries. Other risk factors for HCC are chronic HBV infection and co-infections HBV/HCV, HIV/HCV and HBV/HDV. In the first contribution on HCC, Mario Mondelli (University of Pavia-Fondazione IRCCS Policlinico “San Matteo”, Pavia, Italy), described the viral and cellular oncogenic pathways involved in the development of HCC, focusing on the importance of surveillance that might allow an early diagnosis of HCC in patients with defined risks and consequently reduced mortality. Antiviral treatment can reduce, but not eliminate, the risk of HCC in HBV/HCV infections. A 17 year prospective cohort study coordinated in Pavia [10] has demonstrated conclusively that HCV genotype 1b was associated with a significant higher risk of developing HCC.

In the other contribution Camillo Porta (Fondazione IRCCS Policlinico “San Matteo”, Pavia, Italy) illustrated the molecular pathogenesis of HCC by showing the most relevant pathways, focusing on angiogenesis and the mechanisms of deregulation of proliferation and survival. Several molecularly target therapies are now available, capable of interfering with these pathways at various levels, including sunitinib, bevacizumab, erlotinib, gefitinib and cetuximab, and promising new drugs such as everolimus, and the Met inhibitor ARQ-187. Presently sorafenib, an oral inhibitor of the serine/threonine kinase Raf-1 and of several tyrosine kinases involved in angiogenesis (VEGFR2-3), is the only approved drug. A multicenter study [11], with a relevant Italian contribution, showed that sorafenib prolonged median survival and the time to progression by 3 months in patients with advanced HCC. An important observation [12] is that sorafenib was particularly active in HCV infected patients, while decreasing HCV viral load.

As previously discussed for cervical cancer, miRNAs may be involved in hepatocellular carcinoma as well. The group of Palù evaluated miRNAs expression in relation with the degree of fibrosis, in 40 chronic hepatitis/cirrhosis patients. Results showed that some miRNAs were up-regulated, whereas other miRNAs appeared down-regulated. Relevant miRNAs had several targets that were involved in some biological pathways that promote collagene synthesis, cell proliferation, migration, and epithelial to mesenchimal transition, suggesting a role in viral hepatitis/cirrhosis progression. In a second set of experiments, miRNAs expression was evaluated in 18 HCC patients. Results showed that some of the miRNAs deregulated in advanced grade fibrosis were deregulated in HCC too, suggesting a link between fibrosis and cancer. Future studies will address the role of miRNAs as prognostic indicators or diagnostic markers for HCC.

## Other solid tumors with possible viral pathogenesis, including Merkel cell carcinoma, colorectal cancer, mesothelioma and brain tumors

The first speaker, Mauro Tognon (University of Ferrara, Italy), focused the attention on personal data on polyomaviruses, brain tumors and Merkel cell carcinoma. SV40, a potent transforming polyomavirus, was introduced in the human population about 50 years ago by contaminated polio vaccines, since then its role in the pathogenesis of human brain tumors, mesothelioma and colon cancer has been periodically claimed. SV40 Tag-coding sequences were found in various histotypes of human brain tumors, including 83% of choroid plexus papillomas, 73% of ependymomas, 47% of astrocytomas and 33% of glioblastomas [13,14]. Expression of SV40 early region RNA was also investigated, and 78% of glioblastoma multiforme cell lines positive for SV40 DNA were found also to express SV40 early region sequences. The numbers of viral DNA copies detected by quantitative PCR were very small, with an estimate of 1 copy per 100 cells.

Merkel cell carcinoma is a rare aggressive human skin cancer, with a high mortality rate and an increasing incidence, which is found prevalently in immunocompromised hosts, such as transplant recipients, HIV patients, and subjects with chronic lymphocytic leukemia. Recently, sequences from a new polyomavirus, named Merkel polyomavirus (MCPyV), were found integrated in the genome of a high proportion of Merkel cell carcinoma primary samples. Clonal integration and the deletions and/or mutations observed in the T antigen gene have suggested a direct oncogenic role for MCPyV [15,16]. Tognon presented two collaborative studies aimed to test MCPyV seroprevalence in a general European population and in patients with Merkel cell carcinoma [17,18]. MCPyV viruslike particles (VLPs) were produced in baculovirus cells and these were used to set up an ELISA test for identification and quantification of serum antibodies to MCPyV. MCPyV antibodies were detected in 100% of patients with Merkel cell carcinoma, and in approximately 85% of controls [18]. Similarly to SV40, the new polyomavirus was detected in buffy coats from healthy blood donors [19], with a prevalence of 22%, indicating that exposure to MCPyV is very common.

The session proceeded with a contribution on mesothelioma and gastrointestinal cancers. Manola Comar (University of Trieste, Italy) discussed the SV40 role in the pathogenesis of mesothelioma, as a possible cofactor in the cancerogenetic process. Northeastern Italy may be considered an hyperendemic area for mesothelioma, in association with a massive occupational exposure to asbestos. In a first retrospective Italian study aimed to investigate the presence of SV40 in malignant and normal tissues from mesothelioma patients of this area, Comar and collaborators [20] detected SV40 DNA in approximately 3/19 tumors (15.8%, all positive for asbestos exposure), 3/18 liver and 1/8 of kidney tissues from the same patients. SV40 viral loads were higher in mesothelioma than in normal tissues. These results showed for the first time that SV40 sustained infections in multiple tissues in malignant mesothelioma patients. The association of SV40 with asbestos exposure was confirmed in a larger study based on 63 cases of incident malignant mesothelioma (Comar et al., unpublished observations). These results suggest further investigation to clarify the role of SV40 in the asbestos-dependent transformation process.

Because of localization, providing exposure to external environment, colon cancer may be a target for viral oncogenesis, and this is area of active investigation. Comar presented two studies on polyomaviruses and colon cancer. In the first case–control study [21], DNA from fresh biopsies from 94 cancer patients and 91 control subjects were investigated for SV40, JCV and BK sequences. No DNA from JCV and BK were detected either in patients or controls. SV40 large T antigen fragments were found in colon cancer tissues with a prevalence of 6.4%. The second case–control study [22] was conducted on 64 cases of colon cancer and focused on the recently discovered polyomavirus, MCPyV. Viral sequences were detected with a similarly low prevalence, with no difference between cases and controls (6.3% and 8.8%, respectively). In either study, blood samples were negative. Collectively, these studies demonstrate the existence of SV40 and MCPyV sequences, but no JCV and BK, with low prevalence rates in tumor tissues from colon carcinoma.

EBV has an important role in the pathogenesis of some solid tumors too, namely EBV-related nasopharingeal carcinoma and a variant of gastric cancer, comprising approximately 15% of all gastric cancers. In his talk on EBV-related solid cancers and relative immunotherapy approaches, Fausto Baldanti (Fondazione IRCCS Policlinico “San Matteo”, Pavia, Italy) reported an interesting case of EBV-Smooth Muscle Tumor (SMT) in the context of kidney transplantation successfully treated by minimizing immunosuppression [23], providing the proof of principle that tapering immunosuppression is feasible, successful and safe in patients with non aggressive EBV-SMT. Active front-line therapies, such as the infusion of EBV-specific T cells, antiviral therapies and surgical excision, should be reserved for clinically aggressive tumors or patients at high risk of acute and chronic allograft rejection.

In an Italian retrospective study [24] on post-transplantation proliferative disorders (PTLD) and EBV DNAemia in a cohort of 137 patients receiving lung transplantation, a similar case of EBV-related leiomyosarcoma occurred. In this instance, autologous EBV-specific CTL were used to induce remission of the tumor, giving proof that cell therapy approaches are potentially active in the context of EBV-related solid tumors as well.

## Prevention and therapy

Franco Buonaguro (National Cancer Institute, IRCCS Fondazione “G. Pascale”, Napoli, Italy) introduced the section summarizing features of HPV virus replication, transmission, and immune response. Then the speaker focused the attention on HPV vaccines. Target of preventive vaccines is neutralization of virus before infection of basal keratinocytes. Actual vaccines are based on recombinant L1 protein, a major capsid protein, which is characterized by self assembly to form virus-like particles *in vitro*. Quadrivalent HPV (type 6, 11, 16, 18, from MERCK) and bivalent HPV (type 16, 18, from GSK) L1 VLP vaccines are available, and their efficacy was demonstrated in phase III trials. Results from a phase II randomised placebo controlled trials of a quadrivalent HPV [25] and bivalent HPV L1 VLP vaccines [26] demonstrated efficacy in prevention of incident and persistent cervical infections with HPV-16 and HPV-18, and associated cytological abnormalities and lesions. Protection appears to last for 4–5 years. In a IARC Working Group meta-analysis [27] HPV-16, 18, 45, 31, 33, 35, 52, 58 were the most common HPV types found in invasive cervical cancer. The risk classification of the different HPV types is important for screening and vaccine development, and it’s an open debate, since the oncogenic potential of many virus types is yet to be defined. Another open issue that may have a great impact on the virus transmission to women is HPV vaccination of men, given the role of carrier. To test for the prevalence of HPV infection in immunocompromised patients in Italy, the presence of HPV infection was investigated in urine samples of 88 male patients with renal transplants [28]. The results showed that 48.9% of the samples were positive for HPV sequences, with high prevalence of HPV-16 (53.5%). These results suggested that HPV-16 based preventive or therapeutic vaccine may be effective for prevention and treatment of HPV-related neoplasia in this group of immunocompromised patients.

As regards new HPV vaccines technologies, research is focused on the production of capsomers and in low-cost synthesis. Vaccine production in other systems, such as in plants will permit safer and cheaper synthesis of viral molecules for the developing world. The author is actively pursuing vaccine development in tobacco plants. Therapeutic vaccines are also under development, specifically based on several long peptides derived from proteins E6 and E7. While these strategies have proven to be successful in animal models of HPV neoplasia (mice and rabbits), ways to improve the T helper 1 response are being investigated, including the use of dendritic cells exposed to viral peptides *ex vivo*.

The role of antiviral therapy in HCC prevention was introduced by Raffaele Bruno (Fondazione IRCCS Policlinico “San Matteo”, Pavia, Italy) who presented a collaborative retrospective study of the Italian Association for the Study of the Liver (AISL) [29] on the impact of sustained virological response (SVR) on the long-term outcome of 848 patients with HCV-related cirrhosis treated with IFNα. The results showed that the patients with SVR had a lower risk of liver-related complications and a reduction in liver-related mortality. Another prospective study from Bruno et al. [30] conducted on 352 patients with compensated HCV-induced cirrhosis, confirmed that the achievement of SVR was associated with a reduction of decompensation rate and mortality. These studies also showed that patients with SVR had a lower risk of HCC development. A further study [31] investigated the impact of antiviral therapy in decompensated HCV-related cirrhotic patients. The results showed that the achievement of a SVR in these patients reduced disease progression and may be life-saving. However, SVR rates were significantly lower in patients with advanced fibrosis [32]. SVR rate is also associated with viral genotype. SVR is higher in genotype 2 patients, and so these patients have an advantage in the treatment response [33].

Cell-based immunotherapy against virus-mediated cancers was discussed by Patrizia Comoli (Fondazione IRCCS Policlinico “San Matteo”, Pavia, Italy). The investigator illustrated the last 15 years experience on cell therapy for EBV-related PTLD in immunocompromised patients [34]. Cell therapy was based on generation of EBV-specific CTLs that were used in their experience after preemptive rituximab failure. In high-risk cohorts, such as haplo-HSCT recipients, infusion of donor EBV-specific CTLs contributed to a significant improvement of outcome [35], and induced viral DNA clearance and remission of PTLD in all treated patients [36]. More recently this cell therapy was applied to EBV-related solid tumors, such as nasopharyngeal carcinoma (NPC), in particular in the subset of patients that relapsed after chemo-radiotherapy treatment. Data from the first study conducted [37] showed that successful autologous cell therapy was typically observed in patients with high titers of LMP2-specific responses, with disease control in approximately 50% of patients. With the aim to potentiate *in vivo* T-cell expansion, lymphodepletion was employed before high-dose EBV-specific CTLs in eleven EBV-related NPC, but results were comparable [38]. These preliminary data constituted the ground for an ongoing preclinical study, presented by Comoli, on the production and expansion of LMP2-specific CTL from NPC patients. The conventional procedure of cellular activation is modified by means of LMP2 peptide (15aa) mix. The cell lines obtained by this protocol were successfully used in the treatment of a pediatric patient with refractory NPC. Future studies will attempt to optimize the generation of LMP2-specific CTL in order to increase the rate of T-cells specific for the antigens present on NPC. An important future perspective will be to employ cell therapy in early phases of disease, within a wider protocol, as part of first line treatment or to prevent tumor recurrence in the adjuvant setting.

## Solid tumors in HIV patients

Contributions of the last section were focused on HIV-associated solid tumors. WHO estimates 33.3 million of adults and children infected with HIV in the world, about 820,000 in Europe. In the HAART era, HIV-infected patients survive to older ages, with prolonged exposure to cancer risk factors and, nowadays, non-AIDS Defining Cancers (NADC) represent a much larger fraction of the overall cancer burden. NADC are constituted mainly by Hodgkin’s lymphoma and some solid tumors (lung carcinoma, hepatocarcinoma, GE tumors, head and neck, and anal cancers, skin non melanoma cancers, in decreasing order of frequency) associated with alcohol and smoke exposure, immunedepression, and viral infection (HCV, HPV, HHV-8, MCPyV). Emanuela Vaccher (CRO National Cancer Institute, Aviano, Italy) analyzed data of a recent record-linkage study between the Italian AIDS registry and the Italian cancer registries [39,40]. This study allowed to obtain the standardized incidence ratio (excess risk compared to a general population matched for age and sex, SIR) of cancer in 21,951 AIDS cases reported in Italy between 1986 and 2005, before and after the introduction of HAART. Results showed interesting changes in the cancer pattern after the introduction of HAART in 1996. SIR for Kaposi sarcoma (KS) and non-Hodgkin lymphoma greatly decreased in post-HAART era (although the risk for these cancers remains substantially higher for HIV patients), whereas SIR for invasive cervical cancer, an AIDS defining cancer, were substantially stable, at high levels, in the two periods analyzed. From analysis of the epidemiological data, it is clear that the risk for most NADC were already high in the pre-HAART, with no significant increase in the HAART period, except for HCC (SIR = 6.4) [39]. By analysis of the overall Incidence Rate Ratio (IRR), no significant differences in NADCs emerged after the HAART introduction [40]. Data showed that HCC and lung cancer were the only tumors with increased incidence in the last years in Italy (4.6 and 1.8 times, respectively). An interesting data to investigate further is the impact of aging in the risk of NADCs development of HIV-infected people. Age-specific incidence rates for NADCs in AIDS patients were higher than the corresponding curve in the general population below 45 years of age in pre-HAART and below 55 years of age in HAART era. The analyses of 595 HD cases and 550 solid tumors cases in the CRO-GICAT data base (06/2010) indicated that the major characteristics of HIV-infected patients with solid non-AIDS defining malignancies, compared to the general population were: middle-age groups, more advanced disease stages III-IV (49-93%), and poorer prognosis. National guidelines about HAART administration and diagnostic/clinical approach of HIV-1 patients, introduced some important concepts: NADC patients must be treated with chemotherapy and HAART independently by CD4 count and HIV-RNA; antineoplastic therapy has only support finality in patients without HIV therapeutic chance.

Attention was then focused on HCC and HIV. HCC is an increasing cause of mortality in HIV-infected patients in the HAART era; the risk of HCC is increased in HIV patients (SIR = 3-19). Furthermore, HIV is able to accelerate the evolution process of fibrosis/cirrhosis. In the Italian cohort, 1 year survival of 28% in HIV-positive patients versus a survival of 76.2% in HIV-negative patients has been reported. Antonello Malfitano (Fondazione IRCCS Policlinico “San Matteo”, Pavia, Italy), presented some contributions that explain the main features, clinical presentation and outcome of HCC in HIV-infected patients. The first study presented [41] was based on 41 HIV/HCC patients, that were identified from the GICAT case series in the period 1995–1998. Results showed that in HIV-infected patients liver disease was significantly more advanced at presentation, with higher prevalence of multifocal and infiltrating lesions. Only three variables were significantly more frequent in HIV-positive patients compared with uninfected patients: a higher rate of chronic hepatitis C, a lower prevalence of heavy alcohol intake and a higher frequency of infiltrating lesions and metastasis at first HCC diagnosis. Further aspects to be highlighted were the shorter interval between the estimated date of first HCV exposure and first diagnosis, and the younger age of HIV-positive patients (median age at first diagnosis, 42 years) compared with HIV-negative HCC patients. The process of hepatocarcinogenesis could be more rapid in HIV/HCV-coinfected patients, and an increasing number of cases should be expected in the next few years. A most recent multicenter observational retrospective comparison of 104 HIV/HCC patients and 484 uninfected HCC patients over 13-year period, 1997–2010, was reported [42]. Results of this study confirmed a significantly shorter survival time in HIV-positive HCC patients, despite better Barcelona Clinic Liver Cancer (BCLC) stage at diagnosis and a similar therapeutic option rate.

Last contribution was focused on AIDS-Kaposi’s sarcoma (AIDS-KS). AIDS-KS is the most aggressive form of KS, and Kaposi’s sarcoma herpesvirus (KSHV, also named human herpesvirus 8, HHV-8) is the infectious agent associated with this neoplasia. AIDS-KS most frequently manifests in a multifocal mucocutaneous distribution involving the face, trunk, genitalia, and/or lower extremities. Visceral sites most commonly include the lung and gastrointestinal tract. KS predictive factors are immune system impairment, current CD4+ cell count, current viral load, and absence of combination antiretroviral therapy (cART) [43]. KS risk increases as the CD4+ cell count fell, and is associated with high viral load. HAART is the mainstay of AIDS-KS treatment; other therapeutic approaches can be local (radiation, surgical), interferons, and chemotherapy. Renato Maserati (Fondazione IRCCS Policlinico “San Matteo”, Pavia, Italy), presented some studies [44,45] focused on various aspects of HAART therapy, demonstrating that HAART therapy was successful over a prolonged follow-up, well-tolerated and effective approach for KS.

Co-administration of protease inhibitors with anticancer drugs in the management of HIV-related malignancies can cause potential drug-drug interactions. A study [46] conducted at the CRO-National Cancer Institute-Aviano, investigated the effect of lopinavir/ritonavir (LPV/RTV) on the pharmacokinetics of irinotecan (CPT11). Implications of this study were that the exposure to CPT11 was altered in patients under HAART regimens, including LPV/RTV (and potentially other protease inhibitors with enzyme inhibitory properties).

Although KS treatment is based on HAART, in rare cases individuals may experience KS flare during immune reconstitution inflammatory syndrome (IRIS), a heterogeneous and sometimes fatal disorder of immune perturbation after initiation of highly active antiretroviral therapy. Such a flare does not necessarily indicate a poor prognosis. Maserati reported a study [47] based on 9 HIV-infected patients with KS flare after virologic and immunologic response to HAART, and 10 cases identified by computerized search of the medical literature. Systemic chemotherapy, administered in 10 of 19 cases, led to tumor regression. Even for those patients with rapidly symptomatic KS, early systemic chemotherapy was effective in suppressing IRIS-associated flare.

## The Italian group on viral oncology of solid tumors

The long standing interest and expertise on viral oncology of the several Italian groups participating at the Pavia meeting prompted the organizers to propose a multidisciplinary scientific association with the primary aim to potentiate collaboration throughout the country on basic research, epidemiology, translational and clinical studies on viruses and solid cancer. At the end of the meeting, speakers held a round table discussing these points and expressed intent to participate as investigators at the proposed association.

## Competing interests

The authors declare that they have no competing interests.

## Authors’ contributions

VP and MR wrote the manuscript. All authors read and approved the final manuscript.
